# Transcriptional network analysis of human astrocytic endfoot genes reveals region-specific associations with dementia status and tau pathology

**DOI:** 10.1038/s41598-018-30779-x

**Published:** 2018-08-17

**Authors:** Matthew J. Simon, Marie X. Wang, Charles F. Murchison, Natalie E. Roese, Erin L. Boespflug, Randall L. Woltjer, Jeffrey J. Iliff

**Affiliations:** 1 Department of Anesthesiology and Perioperative Medicine, Oregon Health & Science University, Portland, OR USA; 2 Neuroscience Graduate Program, Oregon Health & Science University, Portland, OR USA; 3 Department of Neurology, Oregon Health & Science University, Portland, OR USA; 4 Department of Pathology, Oregon Health & Science University, Portland, OR USA; 5 Knight Cardiovascular Institute, Oregon Health & Science University, Portland, OR USA

## Abstract

The deposition of misfolded proteins, including amyloid beta plaques and neurofibrillary tangles is the histopathological hallmark of Alzheimer’s disease (AD). The glymphatic system, a brain-wide network of perivascular pathways that supports interstitial solute clearance, is dependent upon expression of the perivascular astroglial water channel aquaporin-4 (AQP4). Impairment of glymphatic function in the aging rodent brain is associated with reduced perivascular AQP4 localization, and in human subjects, reduced perivascular AQP4 localization is associated with AD diagnosis and pathology. Using human transcriptomic data, we demonstrate that expression of perivascular astroglial gene products dystroglycan (*DAG1*), dystrobrevin (*DTNA*) and alpha-syntrophin (*SNTA1*), are associated with dementia status and phosphorylated tau (P-tau) levels in temporal cortex. Gene correlation analysis reveals altered expression of a cluster of potential astrocytic endfoot components in human subjects with dementia, with increased expression associated with temporal cortical P-tau levels. The association between perivascular astroglial gene products, including *DTNA* and megalencephalic leukoencephalopathy with subcortical cysts 1 (*MLC1*) with AD status was confirmed in a second human transcriptomic dataset and in human autopsy tissue by Western blot. This suggests changes in the astroglial endfoot domain may underlie vulnerability to protein aggregation in AD.

## Introduction

Aggregation of mis-folded proteins is a central hallmark of numerous neurodegenerative diseases. For example, Alzheimer’s disease (AD) is characterized histopathologically by the presence of extracellular plaques made up of amyloid β (Aβ) and intracellular neurofibrillary tangles (NFTs) comprised of hyperphosphorylated versions of the microtubule-associated protein tau^1^. Aβ, an extracellular peptide released through the sequential cleavage of the transmembrane amyloid precursor protein (APP), is produced within the brain throughout life in response to synaptic activity^2^. Radiotracer studies carried out in human subjects demonstrate that the slowing of Aβ clearance, rather than an increase in the rate of Aβ production, occurs in both the aging and AD brain^3,4^. It remains unknown, however, what cellular and molecular changes in the aging and AD brain slow Aβ clearance and render it vulnerable to Aβ plaque formation.

The basis for tau aggregation in NFTs remains similarly unclear. Recent experimental studies suggest that tau aggregates propagate in a prion-like manner through the extracellular compartment between neighboring or synaptically connected neurons, potentially accounting for the stereotyped neuroanatomical progression of neurofibrillary pathology throughout the course of AD^5,6^. *In vivo* microdialysis experiments in mice demonstrate that tau is released into the interstitium of the healthy young brain in response to excitatory synaptic activity^7^. Whether changes in the dynamics of extracellular tau underlie the development of NFTs in the aging and AD brain likewise remains unclear.

The glymphatic system is a brain-wide network of perivascular pathways that facilitates the exchange of interstitial and cerebrospinal fluid (CSF), supporting the clearance of Aβ and tau from the brain interstitium. Glymphatic function, including Aβ clearance, is dependent upon the expression of the astroglial water channel aquaporin-4 (AQP4) that is localized primarily to perivascular astrocytic endfeet ensheathing the cerebral vasculature^8^. *Aqp4* gene deletion in mice slows Aβ clearance^8^ and promotes Aβ plaque formation in a mouse model of AD^9^, and exacerbates pathological tau phosphorylation in an experimental model of traumatic brain injury^10^. In the aging and post-traumatic rodent brain, impairment of glymphatic function is associated with the loss of perivascular AQP4 localization^10,11^.

Glymphatic function has been visualized clinically only on a limited basis^12,13^, and it is not yet known whether age-related impairment in glymphatic function contributes to the development of AD pathology. However, a recent study in human post-mortem tissue demonstrated that loss of perivascular AQP4 localization is associated with AD status, and with both Aβ plaque burden and neurofibrillary pathology^14^. In the present study, we use human transcriptomic data from aged cognitively-intact and subjects with dementia to demonstrate that changes in the expression of components of the dystrophin-associated complex (DAC), the multi-protein complex that anchors AQP4 to the astrocytic perivascular endfoot, are associated with dementia status and tau pathology in the human cortex. Using Weighted Gene Co-expression Network Analysis (WGCNA), we identify a cluster of 11 astrocytic genes that have similar gene expression profiles with DAC genes and *AQP4* in the aging brain, and whose expression levels predict dementia status and are strongly associated with temporal cortical tau pathology. Lastly, we confirm the associations between the newly identified genes and AD pathology at both the gene expression and protein levels in independent human AD cohorts. These findings suggest that changes in perivascular astroglial function may be one factor contributing to the development of AD pathology.

## Results

To evaluate the relationship between perivascular astroglial gene expression and AD pathology in the aging and AD brain, we utilized publicly available data from the Allen Brain Institute Aging, Dementia and TBI study (Supplementary Table 1)^15^. Demographic data on study subjects is provided in Table 1. The study included RNA sequencing (RNAseq) and clinical data from 57 subjects that remained free from dementia through the course of the community-based Adult Changes in Thought (ACT) study and 50 subjects with a clinical diagnosis of dementia. Of these, 22 met NINDS-ARDA Alzheimer’s criteria for ‘Probable AD’, 21 met criteria for ‘Possible AD’ and 7 were ‘Unlikely AD’ (including 3 cases with clinical diagnosis of vascular dementia). Ages were well matched between Non-Dementia and Dementia groups and expected differences in gender makeup (more women among dementia vs. dementia free subjects), APOε4 allele carrier status (more APOε4 allele carriers among subjects with dementia) and educational attainment (lower levels of educational attainment among subjects with dementia) were observed. As anticipated, Aβ plaque burden and NFT severity, measured by the Consortium to Establish a Registry for Alzheimer’s disease (CERAD) score and Braak stage, respectively, were greater among dementia versus dementia-free subjects.

**Table 1:** Subject Demographic Data.

	n	Number males (%)	APOε4 carriers (%)	Education	CERAD Score	Braak Stage
**Non-Dementia**	57	36 (63.2)	7 (12.3)	15 (12.5, 16)	1 (1, 2)	3 (2, 4)
77–89 yrs	31	24 (77.4)	4 (12.9)	16 (14, 17)	1 (1, 1)	3 (1, 3)
90+ yrs	26	12 (46.2)	3 (11.5)	14 (12, 16)	1 (1, 2)	3 (2, 4.25)
**Dementia**	50	27 (54.0)	13 (26.0)	13 (12, 16)	**2** (**0**.**75**, **3**)*	**4** (**3**, **6**)***
77–89 yrs	26	13 (50.0)	8 (30.8)	**12**.**5** (**12**, **16**)*	1.5 (0, 3)	**4** (**2**, **6**)**
90+ yrs	24	14 (58.3)	5 (20.8)	13.5 (12, 15.75)	**2**.**5** (**1**.**25**, **3**)*	**5** (**3**.**25**, **5**.**75**)**

*P < 0.05, **P < 0.01, ***P < 0.001 vs. corresponding Non-Dementia group. Statistical associations determined by Mann-Whitney *U* test. Education, CERAD Score, Braak Stage include median (1^st^ and 3^rd^ quartile) values.

### *AQP4* expression is associated with increased Aβ burden in the parietal cortex

RNAseq-based profiling of the transcriptome was performed in the tissue from temporal (TCX) and parietal cortex (PCX), hippocampus (HIP) and frontal white matter (FWM). Two techniques measured Aβ burden and tau pathology in each region: immunohistochemistry (6E10 and AT8 antibodies, respectively) and quantitative Aβ_1–40_, Aβ_1–42_, pTau-181 (P-tau) and total tau Luminex assays.

**Figure 1:**
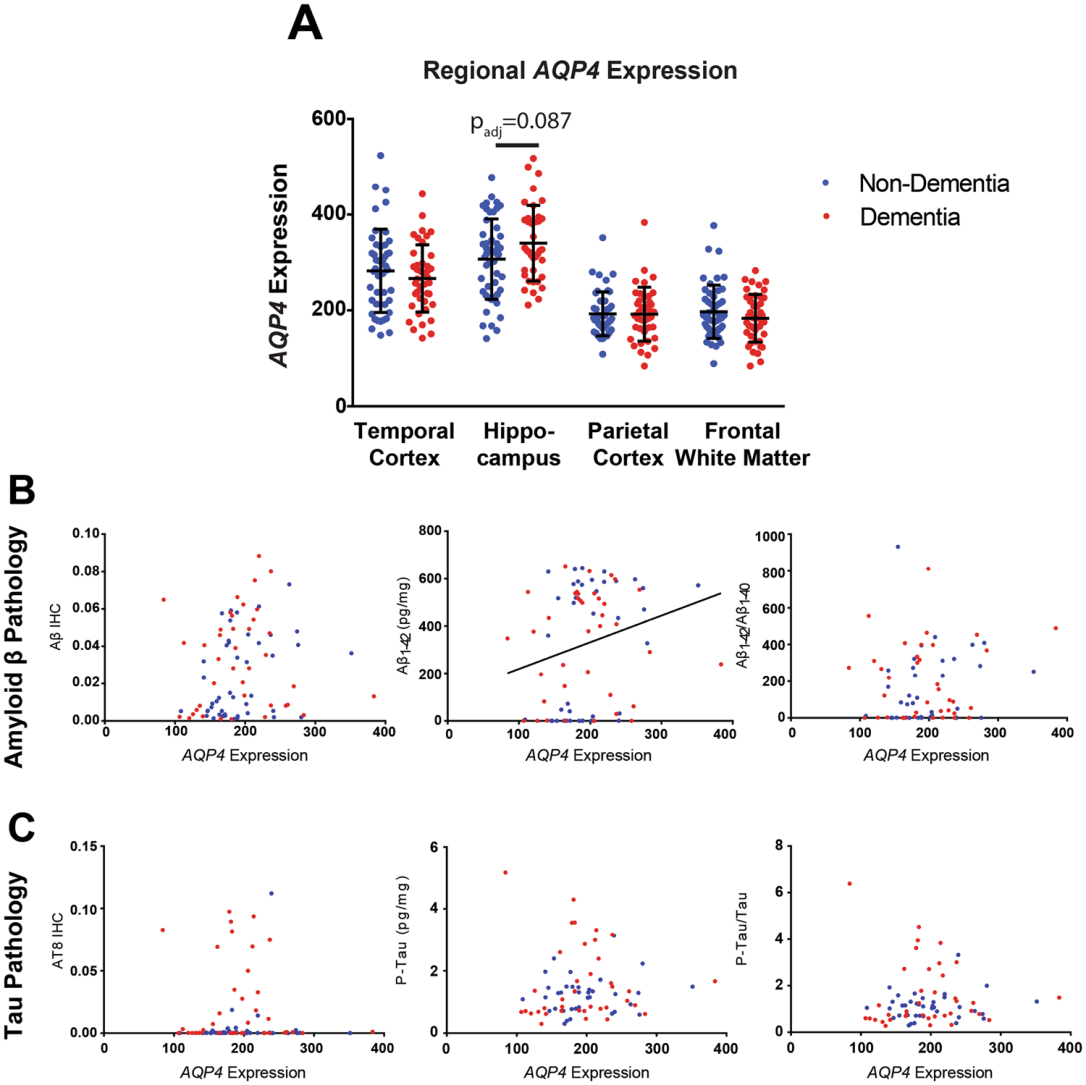
Elevated *AQP4* gene expression is associated with parietal cortical Aβ. (**A**) Expression levels of *AQP4* were quantified across 4 brain regions, TCX, HIP, PCX, and FWM, in subjects with (blue, n = 57) and without (red, n = 50) dementia. Hippocampus demonstrates a trend towards elevated AQP4 in subjects with dementia (logistic regression, t = −0.947, p_adj_ = 0.087). (**B**) Significant associations between *AQP4* expression with Aβ_1–42_ levels (center, OLS regression, t = 3.29, p_adj_ = 0.028), were detected in PCX. (**C**) No significant association between *AQP4* expression and markers of tau pathology were detected in PCX. The bars in figure (**A**) represent the mean ± the standard deviation (S.D.). The line in figure (**B**) center represents the least squares (ordinary) fit for all data points that show a significant statistical association.

Prior work from our group demonstrated that changes in AQP4 protein expression and localization are associated with Aβ and progression of tau pathology^10,14^. Based on this, we first evaluated the relationship between *AQP4* gene expression and dementia status. When controlling for age, gender, APOε4 and traumatic brain injury status, there was a trend towards greater *AQP4* expression in the HIP of individuals with dementia (t = 1.895, p_adj_ = 0.087), and no statistically significant differences in gene expression were observed across any of the four brain regions (Fig. 1A). We next assessed if transcript level expression of *AQP4* is associated with Aβ and tau pathology. Consistent with previous findings, a significant positive association between *AQP4* expression and Aβ_1–42_ concentration (t = 2.238, p_adj_ = 0.028) was observed in the PCX (Fig. 1B and Table 2). *AQP4* expression was not significantly associated with markers of tau pathology in any region (Fig. 1C, Table 2).

**Table 2:** Association of AQP4 and DAC gene expression with Alzheimer’s pathology.

		*AQP4*	*DAG1*	*DMD*	*DTNA*	*SNTA1*
Temporal Cortex	Aβ IHC	0.525	−0.747	0.181	0.569	−0.150
	Aβ_1–40_ (pg/mg)	1.598	0.515	−0.316	*2*.*277* (*0*.*025*, *0*.*051*)	**2**.**579** (**0**.**011**, **0**.**034**)
	Aβ_1–42_ (pg/mg)	0.811	−0.125	−1.247	1.138	0.106
	Aβ_1–42_/Aβ_1–40_	0.222	−0.727	−1.144	−0.002	−1.480
	AT8 IHC	0.527	−0.005	0.435	−0.109	−0.681
	P-Tau (ng/mg)	1.897	**3**.**290** (**0**.**001**, **0**.**010**)	−0.317	1.971	**2**.**380** (**0**.**019**, **0**.**038**)
	P-Tau/Tau_total_	1.603	**2**.**953** (**0**.**004**, **0**.**023**)	−0.295	1.870	1.948
Hippocampus	Aβ IHC	−0.090	1.182	−1.525	0.523	0.072
	Aβ_1–40_ (pg/mg)	−0.287	0.062	1.273	−0.014	0.612
	Aβ_1–42_ (pg/mg)	−0.013	0.373	−1.503	0.778	1.804
	Aβ_1–42_/Aβ_1–40_	0.079	0.383	−2.067	0.847	1.746
	AT8 IHC	0.643	1.392	−1.198	0.665	−0.171
	P-Tau (ng/mg)	0.883	0.669	0.253	0.857	−0.753
	P-Tau/Tau_total_	1.005	0.520	0.031	0.855	−0.753
Parietal Cortex	Aβ IHC	1.742	0.113	0.606	−0.288	0.864
	Aβ_1–40_ (pg/mg)	1.446	−1.028	1.579	0.534	−0.339
	Aβ_1–42_ (pg/mg)	**2**.**238** (**0**.**002**, **0**.**028**)	−1.281	−0.314	2.021	−1.340
	Aβ_1–42_/Aβ_1–40_	1.268	−0.597	−1.519	1.744	−1.177
	AT8 IHC	0.725	−0.005	−0.884	−0.209	−0.299
	P-Tau (ng/mg)	0.907	1.574	−0.121	−0.803	0.495
	P-Tau/Tau_total_	0.683	1.498	−0.115	−0.729	0.477
Frontal White Matter	Aβ IHC	0.553	0.033	0.149	1.169	0.695
	Aβ_1–40_ (pg/mg)	−0.323	−0.516	−0.368	−0.373	0.434
	Aβ_1–42_ (pg/mg)	−0.097	−1.175	−0.448	0.231	−0.561
	Aβ_1–42_/Aβ_1–40_	0.061	−0.972	−0.251	0.406	−0.820
	AT8 IHC	−0.358	0.148	0.052	0.067	1.521
	P-Tau (ng/mg)	0.568	1.859	−0.127	−*2*.*194* (*0*.*031*, *0*.*061*)	0.545
	P-Tau/Tau_total_	0.555	1.856	−0.110	−1.802	0.684

*t*-statistics for the association from Ordinary Least Squares (OLS) regression corrected for age, APOε4 status and TBI history. P_adj_ < 0.05 encoded in bold. Italics indicate outcomes that were statistically significant prior to, but not after false discovery rate and multiple comparison correction. Values in parenthesis represent the unadjusted (left) and adjusted (right) P-values. Positive t statistics represent a positive association between gene expression and pathology outcomes, while negative statistics represent an inverse association between gene expression and pathology.

### Astrocytic dystrophin-associated complex components are associated with temporal tau pathology

**Figure 2:**
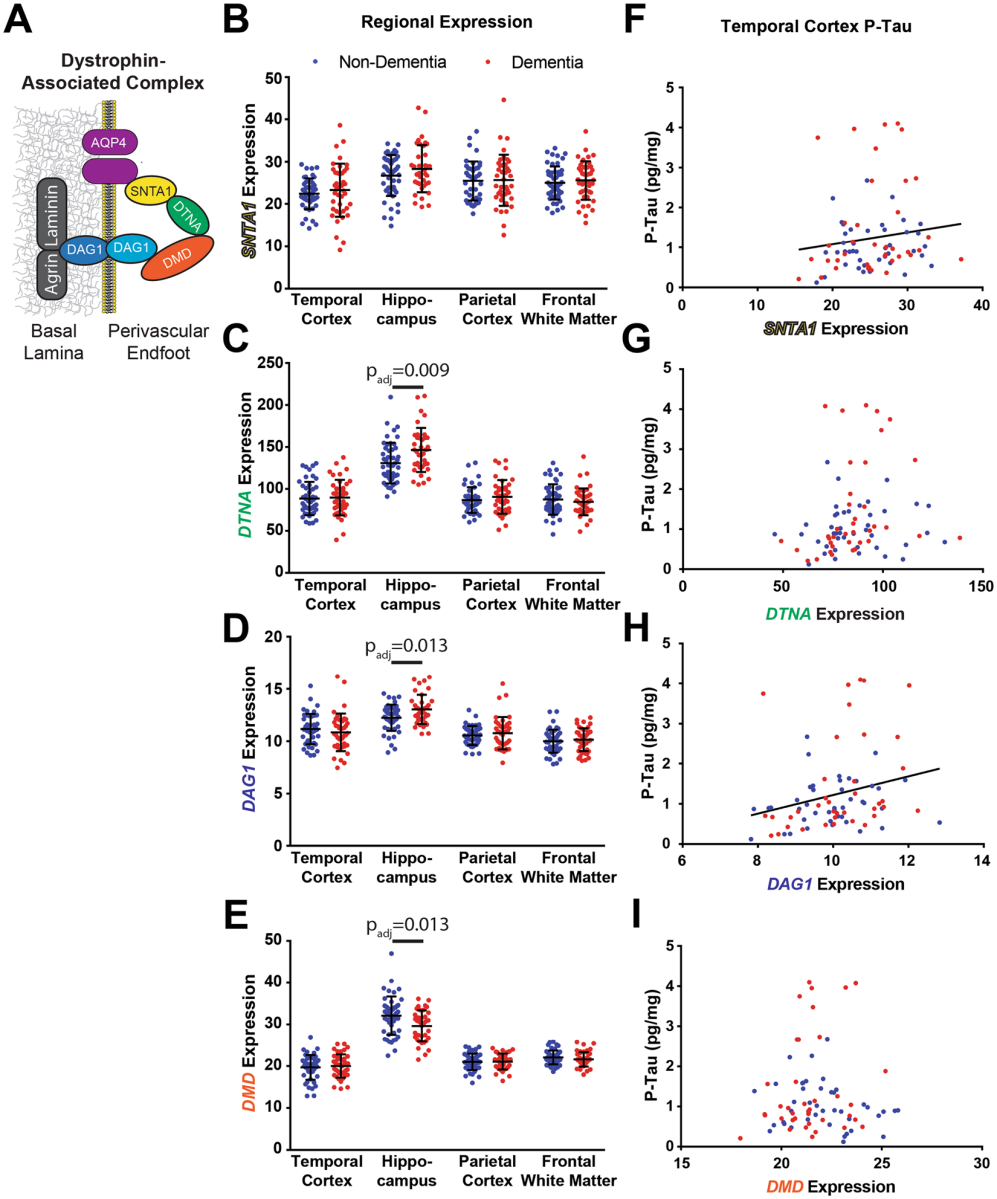
Dystrophin-associated complex (DAC) gene expression is associated with dementia status and temporal cortical P-tau levels. (**A**) Schematic of the DAC at the astrocytic endfoot membrane. Known components of the complex include aquaporin-4 (AQP4), α-syntrophin (SNTA1), dystrobrevin (DTNA), dystrophin (DMD) and dystroglycan (DAG1). (**B**–**E**) Expression levels of the 4 DAC components across the 4 brain regions. *DTNA* and *DAG1* demonstrate significantly elevated expression in the HIP of subjects with dementia (t = 2.731, 2.632; p_adj_ = 0.009, 0.013; respectively), while *DMD* expression is reduced in HIP among subjects with dementia (t = −2.615, p_adj_ = 0.013). (**F**–**I**) Association of DAC gene expression with TCX P-tau levels. *SNTA* and *DAG1* demonstrate associations with tau pathology regardless of dementia status (t = 2.380, 3.29; p_adj_ = 0.038, 0.010; respectively). The bars in figures (**B**–**E**) represent the mean ± the standard deviation (S.D.). The line in figures (**F**,**H**) represent the least squares (ordinary) fit for all data. All statistical associations between gene expression and dementia status assessed using logistic regression. All statistical associations between gene expression and pathology indicators were evaluated using OLS regression.

AQP4 is anchored at the perivascular astrocytic endfoot by the DAC, comprised of dystrophin (*DMD*), dystroglycan (*DAG1*), dystrobrevin (*DTNA*) and α-syntrophin (*SNTA1*), which is necessary for AQP4 localization (Fig. 2A). We evaluated whether hippocampal expression of the four DAC genes share a similar relationship with dementia status and AD pathology as *AQP4*. While *SNTA1* expression did not differ between dementia statuses (Fig. 2B, t = 1.437, p_adj_ = 0.226), both *DTNA* and *DAG1* expression in the HIP were significantly increased among subjects with dementia (Fig. 2C,D, t = 2.731, 2.632; p_adj_ = 0.009, 0.013; respectively). In contrast, *DMD* expression was significantly reduced in the HIP of subjects with dementia (Fig. 2E, t = 2.632, p_adj_ = 0.013). No changes in the expression of *AQP4* (Fig. 1A) nor any of the 4 DAC genes (Fig. 2B–E) in the TCX, PCX or FWM significantly predicted dementia status.

Assessment of the relationship between different DAC gene expression and AD pathology revealed no significant associations with markers of Aβ or phosphorylated tau within the PCX, HIP or FWM (Table 2). However, within the TCX, increased *SNTA1* expression was associated with increased levels of Aβ_1–40_ (t = 2.579, p_adj_ = 0.034). Strikingly, higher expression of *SNTA1* and *DAG1* were both significantly associated with elevated P-tau levels (Fig. 2F,H, t = 3.290, 2.380; p_adj_ = 0.010, 0.039; respectively). *DNTA* and *DMD* did not demonstrate these associations (Fig. 2G,I). While these results support the role of AQP4 as a regulator of Aβ dynamics, they further suggest that other elements of the astroglial perivascular endfoot processes may contribute to the development of tau pathology within the TCX.

### Unbiased identification of candidate genes that may encode elements of the astrocytic endfoot

We next sought to identify candidate genes that may encode proteins involved in perivascular astrocytic endfoot function. To achieve this, we identified genes with similar expression profiles to the ‘established endfoot genes’ including *AQP4* and the DAC component genes. We first confirmed the astrocyte specificity of *AQP4* and DAC gene expression within the brain using two independent single-cell RNAseq datasets: the Allen Brain Institute Cell Types dataset and the Brain RNAseq database (Supplementary Fig. 1 and Supplementary Table 1)^16,17^. With the exception of *DMD*, greater than 50% of total gene expression was derived from astrocytes for the established DAC genes (*SNTA1*, *DAG1*, *DTNA*) and *AQP4*.

**Figure 3:**
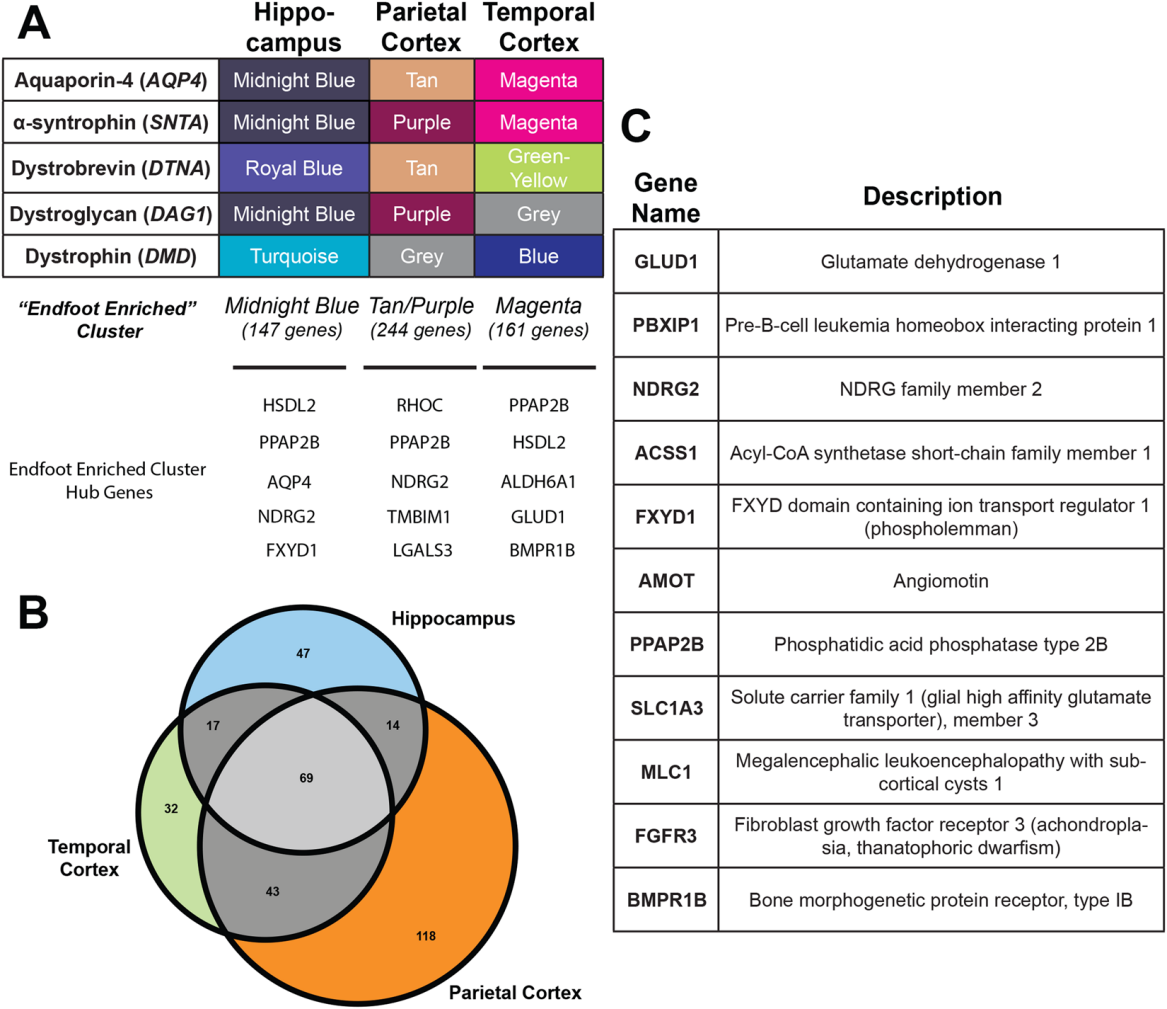
Identification of novel endfoot gene candidates by Weighted Gene Correlation Network Analysis (WGCNA). (**A**) Cluster in which each of the five established endfoot genes assembled for each region. The cluster containing the greatest number of established endfoot genes was defined as the “endfoot enriched” cluster (bottom). Hub genes for each cluster are also identified. (**B**) Gene expression overlap for the “endfoot enriched” cluster across gray matter regions. (**C**) Consensus endfoot candidate genes identified by thresholding for astrocyte specificity (≥50% total expression), co-clustering with DAC genes across multiple brain regions, and high Pearson’s correlation with DAC genes.

To identify novel endfoot candidate genes, we utilized the unbiased, biologically motivated hierarchical clustering method of WGCNA to identify genes with similar human RNAseq expression profiles to the established endfoot genes. WGCNA was performed independently for each of the 4 brain regions: PCX, TCX, HIP and FWM (Supplementary Dataset 1). All regions except for FWM demonstrated co-clustering of the established endfoot genes, and the cluster(s) that contained the greatest number of the endfoot established genes were defined as the “endfoot enriched cluster(s)” (Fig. 3A). Based on the incoherent clustering of DAC genes and *AQP4* in the FWM further analyses focused on the PCX, TCX and HIP regions.

Upon generation of a single WGCNA network for all subjects, 340 genes sorted in the endfoot-enriched cluster in at least one of the 3 gray matter regions (Fig. 3B). 143 of these genes appeared in the endfoot-enriched cluster in more than one region. After applying a stringent gene expression correlation-based thresholding process and filtering out genes that are not predominantly expressed in astrocytes, 11 genes were identified within these clusters that had highly robust correlations with established endfoot genes (*AQP4*, *DTNA*, *SNTA1*, *DAG1* and *DMD*) and demonstrate enriched expression in astrocytes across multiple brain regions (Fig. 3C). To determine the most connected genes within modules, intramodular connectivity was assessed for all genes within the end foot enriched clusters. In all three regions the novel genes were hub genes within the endfoot-enriched clusters (Fig. 3A). Surprisingly, generation of a consensus network across dementia status resulted in less robust clustering of novel genes into the endfoot-enriched module when comparing across brain regions (Supplementary Dataset 1). In HIP, all candidate genes again clustered in the endfoot-enriched module, while in TCX 8 of the 11 genes co-clustered with *SNTA1* and in PCX, there were no consistent clustering patterns among novel genes. These results suggest that associations observed in the HIP and TCX may be particularly robust while associations in PCX may not be as consistent.

Literature review revealed that three of these 11 candidate genes (*MLC1*, *NDRG2* and *SLC1A*) are previously reported components of the perivascular astrocytic endfoot (Supplementary Table 2). Though the present WGNCA was carried out based on 3 brain regions from aged subjects (minimum 77 years), we previously identified 9 of these 11 genes as possible endfoot genes based upon a similar WGCNA analysis of RNAseq data from human brain samples at various developmental time points (Supplementary Table 2)^18^. These 11 gene endfoot candidates thus represent a novel pool of potential regulators of perivascular astrocytic function across aging and disease states.

### Expression of novel endfoot candidate genes is significantly associated with dementia status and tau pathology

**Figure 4:**
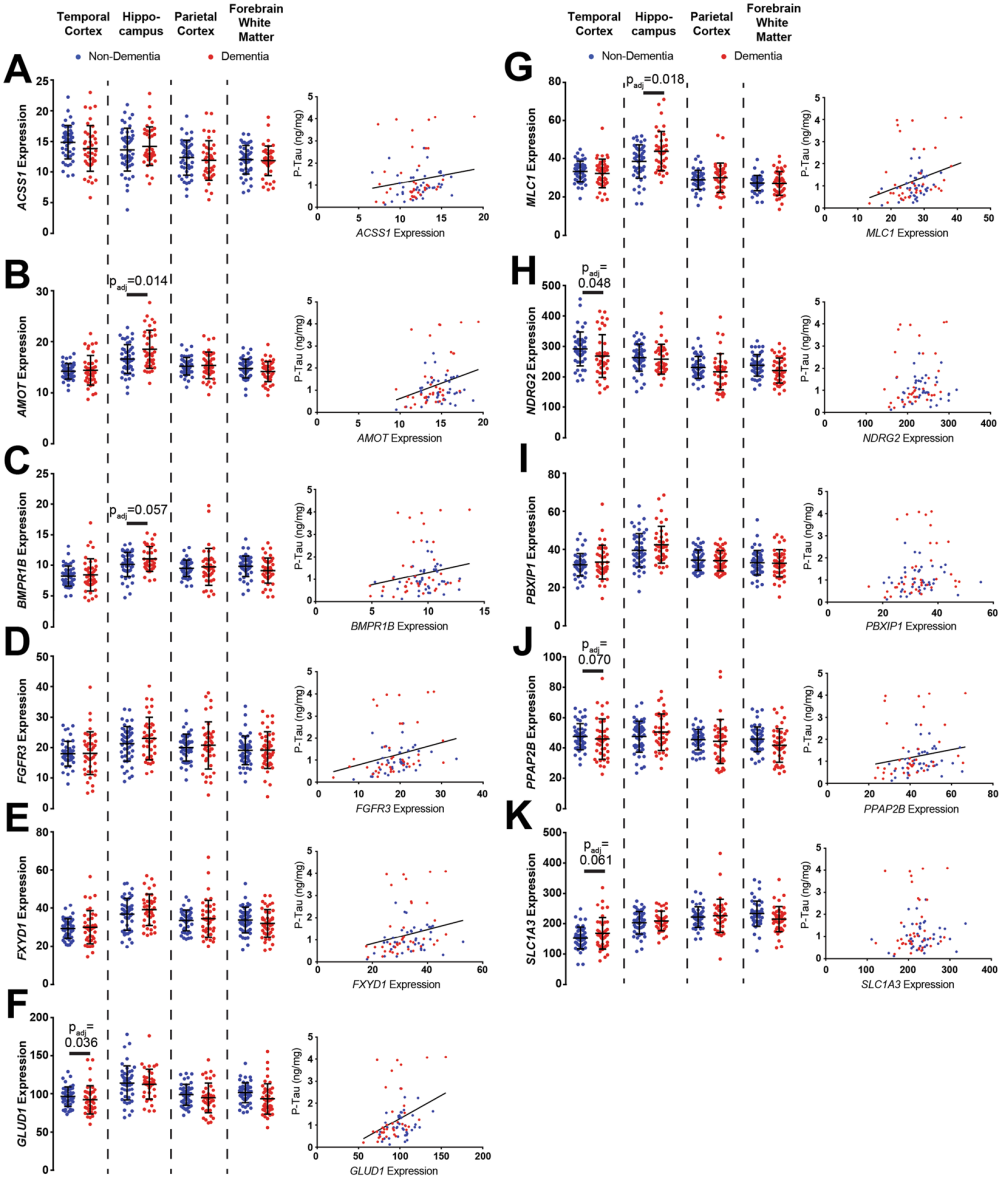
Novel candidate endfoot gene expression is associated with temporal cortical tau pathology and dementia status. For each gene (**A**–**K**, left), expression is quantified for each brain region in cognitively intact subjects (blue, n = 57) and subjects with dementia (red, n = 50). Candidate endfoot gene expression was increased in HIP of subjects with dementia for *AMOT* (**B**, t = 2.606, p = 0.014), and *MLC1* (**G**, t = 2.512, p = 0.018). Reduced expression of *GLUD1* (**F**, t = −2.255, p = 0.036) and *NDRG2* (**H**, t = −2.120, p = 0.048) was observed in the TCX among subjects with dementia. All candidate endfoot genes, with exception of *NDRG2*, *PBXIP1* and *SLCA1A3* exhibited significant associations with P-tau levels (right). This included *ACSS1* (t = 2.573, p = 0.038), *AMOT* (t = 3.549, p = 0.005), *BMPR1B* (t = 2.498, p = 0.047), *FGFR3* (t = 4.196, p < 0.001), *FXYD1* (t = 3.383, p = 0.005), *GLUD1* (t = 3.986, p = 0.001), *MLC1* (t = 4.115, p < 0.001) and *PPAP2B* (t = 2.856, p = 0.028). The bars in figures (**A**–**K**, left) represent the mean ± the standard deviation (S.D.). The line in figures (**A**–**K**, right) represent the least squares (ordinary) fit for all data points that show a significant statistical association. All statistical associations between gene expression and dementia status assessed using logistic regression. All statistical associations between gene expression and pathology indicators were evaluated using OLS regression.

Like the established DAC genes *AQP4*, *DTNA*, *DAG1* and *DMD*, expression of several endfoot candidate genes differed between dementia-free and subjects with dementia (Fig. 4A–K). *AMOT* (Fig. 4B, t = 2.606, p_adj_ = 0.014) and *MLC1* (Fig. 4G, t = 2.512, p_adj_ = 0.018) expression in HIP was significantly greater in subjects with dementia than dementia-free subjects, while *BMPR1B* (Fig. 4C, t = 2.073, p_adj_ = 0.057) exhibited a similar trend that did not remain significant after correction for multiple comparisons. Within TCX, expression of *GLUD1* (Fig. 4F, t = −2.255, p_adj_ = 0.036) and *NDRG2* (Fig. 4H, t = −2.120, p_adj_ = 0.048) were significantly lower in subjects with dementia versus dementia-free subjects. A similar trend was observed for both *PPAP2B* (Fig. 4J, t = −1.986, p_adj_ = 0.070) and *SLC1A3* (Fig. 4K, t = −2.048, p_adj_ = 0.061) in the TCX, although these effects did not survive correction for multiple comparisons.

Pathological analysis revealed a striking association between these endfoot candidate genes and temporal tau pathology. 8 out of 11 genes, including *ACSS1*, *AMOT*, *BMPR1B*, *FGFR3*, *FXYD1*, *GLUD1*, *MLC1*, and *PPAP2B* were each associated with increasing P-Tau levels within the TCX (Table 3). These associations were unique to the genes identified and not seen between P-tau and the endfoot enriched module eigengene (Supplementary Table 3). Upon initial analysis, *PBXIP1* was associated with P-Tau levels in TCX, however this association did not persist following correction for multiple comparisons. Only *NDGR2* and *SLC1A3* did not demonstrate any association with tau pathology. As seen in Table 3, significant associations between increasing gene expression and increasing P-Tau were observed in PCX (*FGFR3*) and FWM (*AMOT*, *FGFR3*). Interestingly, in PCX significant associations between tau pathology and the endfoot enriched cluster eigengene were observed, suggesting other genes may also be of interest that were eliminated based on other thresholding criteria (Supplementary Table 3). There were also significant positive associations between increasing TCX expression of *FXYD1*, *MLC1* and *PBXIP1*, and Aβ_1–40_ levels. These findings suggest that these endfoot candidate genes, like the DAC elements, may play an important role in the development of P-tau aggregation.

**Table 3:** Association of WGCNA-identified gene products with Alzheimer’s pathology.

		*ACSS1*	*AMOT*	*BMPR1B*	*FGFR3*	*FXYD1*	*GLUD1*	*MLC1*	*NDRG2*	*PBXIP1*	*PPAP2B*	*SLC1A3*
Temporal Cortex	Aβ IHC	0.301	0.810	−0.727	0.158	−0.217	1.234	0.069	−1.037	0.032	−0.593	−0.965
	Aβ_1–40_ (pg/mg)	0.902	*2*.*260* (*0*.*026*, *0*.*051*)	*2*.*187* (*0*.*031*, *0*.*061*)	1.312	**2**.**719** (**0**.**008**, **0**.**015**)	2.118	**2**.**719** (**0**.**008**, **0**.**015**)	0.760	**3**.**493** (**0**.**001**, **0**.**001**)	1.656	1.338
	Aβ_1–42_ (pg/mg)	−0.599	0.615	0.039	−1.409	−0.224	−0.351	−1.384	−1.121	0.685	−0.112	−0.423
	Aβ_1–42_/Aβ_1–40_	−1.310	−0.816	−1.199	−**2**.**471** (**0**.**015**, **0**.**030**)	−1.873	−1.534	−**3**.**031** (**0**.**003**, **0**.**006**)	−1.718	−1.441	−1.190	−0.890
	AT8 IHC	0.364	1.078	−0.081	0.199	1.141	0.597	0.931	−1.285	1.843	−0.229	−0.464
	P-Tau (ng/mg)	**2**.**573** (**0**.**012**, **0**.**023**)	**3**.**549** (**0**.**001**, **0**.**001**)	**2**.**498** (**0**.**014**, **0**.**027**)	**4**.**196** (<**0**.**001**, <**0**.**001**)	**3**.**383** (**0**.**001**, **0**.**002**)	**3**.**986** (<**0**.**001**, <**0**.**001**)	**4**.**115** (<**0**.**001**, <**0**.**001**)	1.990	**2**.**463** (**0**.**016**, **0**.**031**)	**2**.**856** (**0**.**005**, **0**.**010**)	1.217
	P-Tau/Tau_total_	*2*.*462* (*0*.*016*, *0*.*031*)	**3**.**397** (**0**.**001**, **0**.**002**)	2.080	**3**.**868** (<**0**.**001**, <**0**.**001**)	**3**.**154** (**0**.**002**, **0**.**004**)	**3**.**467** (**0**.**001**, **0**.**001**)	**4**.**020** (<**0**.**001**, <**0**.**001**)	1.637	*2*.*159* (*0*.*033*, *0*.*065*)	**2**.**390** (**0**.**019**, **0**.**037**)	0.953
Hippo-campus	Aβ IHC	0.300	1.314	0.892	1.052	−0.818	−0.588	0.492	−0.284	−0.312	−0.188	−0.522
	Aβ_1–40_ (pg/mg)	0.401	0.438	−0.370	−0.103	−0.149	−0.593	−0.202	−0.400	−0.076	0.171	−1.559
	Aβ_1–42_ (pg/mg)	0.381	0.463	0.067	−0.206	−0.319	−1.256	0.035	0.043	1.522	0.705	0.097
	Aβ_1–42_/Aβ_1–40_	0.282	0.358	0.192	−0.189	−0.296	−1.163	0.103	0.176	1.676	0.706	0.603
	AT8 IHC	−0.717	0.306	0.115	−0.592	−1.820	−1.838	−1.218	−2.600	0.238	−1.258	0.069
	P-Tau (ng/mg)	−0.301	0.726	1.999	−0.387	−0.098	0.380	−0.814	−0.692	0.512	1.046	0.263
	P-Tau/Tau_total_	−0.229	0.693	*2*.*157* (*0*.*034*, *0*.*066*)	−0.527	−0.077	0.423	−0.902	−0.714	0.576	1.250	0.322
Parietal Cortex	Aβ IHC	1.302	0.706	0.454	1.121	0.537	1.056	0.919	0.413	0.651	0.906	−1.086
	Aβ_1–40_ (pg/mg)	0.009	0.317	−0.075	−0.171	−0.349	1.038	−0.033	−0.568	−0.316	−0.043	−1.340
	Aβ_1–42_ (pg/mg)	−1.709	0.076	0.386	−1.229	−0.046	0.100	−1.050	−1.117	−0.099	0.055	0.358
	Aβ_1–42_/Aβ_1–40_	−1.843	−0.155	0.470	−1.186	0.211	−0.664	−1.101	−0.766	0.130	0.091	1.390
	AT8 IHC	−0.987	0.393	−0.550	−0.352	−0.250	−0.766	−0.201	−**2**.**671** (**0**.**021**, **0**.**041**)	−0.092	−1.107	−1.079
	P-Tau (ng/mg)	0.749	2.113	0.916	**2**.**735** (**0**.**020**, **0**.**015**)	2.033	2.069	**2**.**376** (**0**.**001**, **0**.**038**)	−0.429	0.075	1.049	−1.249
	P-Tau/Tau_total_	0.612	*2*.*128* (*0*.*036*, *0*.*071*)	0.738	**2**.**641** (**0**.**028**, **0**.**019**)	1.836	1.788	*2*.*233* (*0*.*001*, *0*.*054*)	−0.583	0.043	0.866	−1.318
Frontal White Matter	Aβ IHC	−0.037	0.279	1.153	0.613	1.267	0.488	0.431	0.256	1.637	1.226	**3**.**086** (**0**.**003**, **0**.**005**)
	Aβ_1–40_ (pg/mg)	0.889	1.656	0.314	1.597	1.230	1.013	0.793	0.047	0.245	0.525	0.274
	Aβ_1–42_ (pg/mg)	−0.360	0.211	−0.931	−1.093	−0.643	−0.458	−1.565	−1.122	−0.286	−0.560	−0.079
	Aβ_1–42_/Aβ_1–40_	−0.898	−0.684	−1.156	-*2*.*040* (*0*.*044*, *0*.*087*)	−1.330	−1.088	−2.125	−1.263	−0.422	−0.885	−0.213
	AT8 IHC	−1.281	−0.155	−1.210	−0.168	−0.162	−1.279	−0.127	−1.910	0.008	−1.362	−0.432
	P-Tau (ng/mg)	0.440	*2*.*291* (*0*.*024*, *0*.*047*)	0.292	**4**.**015** (<**0**.**001**, <**0**.**001**)	1.045	1.937	0.803	−0.812	0.307	1.765	−0.726
	P-Tau/Tau_total_	0.290	**2**.**566** (**0**.**012**, **0**.**023**)	0.399	**4**.**239** (<**0**.**001**, <**0**.**001**)	1.202	1.947	1.050	−0.968	0.420	1.666	−0.628

*t*-statistics for the association from Ordinary Least Squares (OLS) regression corrected for age, APOε4 status and TBI history. P_adj_ < 0.05 encoded in bold. Italics indicate outcomes that were statistically significant prior to, but not after false discovery rate and multiple comparison correction. Values in parenthesis represent the unadjusted (left) and adjusted (right) P-values. Positive t statistics represent a positive association between gene expression and pathology outcomes, while negative statistics represent an inverse association between gene expression and pathology.

### Validation of protein level expression changes for a subset of novel gene candidates

The Allen Aging, Dementia and TBI cohort was generated to assess age-related changes in brain gene expression and neuropathology while directly controlling for the effects of TBI, but not AD. Additionally, this dataset does not include protein expression data for AQP4, DAC gene products or the candidate endfoot proteins. To address these issues, we sought to validate the identified associations in two independent AD cohorts, both at the gene and protein expression levels. We selected four genes from the pool of known and novel candidate endfoot genes: *AQP4*, *DTNA*, *MLC1* and *FXYD1*. *AQP4* was selected due to its established role in Aβ dynamics^8,9^ and associations with AD status and pathology^14^. From the DAC complex, we selected *DTNA* based on the robust associations with dementia status and P-tau pathology. *MLC1* and *FXYD1* were chosen from the larger pool of novel gene candidates based on the associations identified with dementia status and tau pathology. Like *DTNA*, hippocampal *MLC1* expression robustly predicts dementia status while TCX expression is strongly associated with P-tau levels. In contrast, *FXYD1* expression does not predict dementia status, but is strongly associated with temporal cortical P-tau levels.

We first validated the observed gene expression changes in the Hisayama Study Microarray Dataset (Supplementary Table 1), an independently generated AD gene expression cohort. This dataset contains microarray data from 47 non-AD and 32 AD subjects ranging in age from 54 to 105 years of age. Samples were collected from three different grey matter regions including HIP, frontal cortex (FCX) and TCX. Gender distributions within each group were approximately even, however there is a significant difference in median age between AD (median = 91 years, Interquartile range (IQR) = 7 years) and non-AD subjects (median = 80 years, IQR = 9 years). In contrast to non-AD subjects selected for Western blot analysis from the Oregon Brain Bank, which were selected based on the absence of clinic-pathological diagnosis of vascular or mixed dementia, the Hisayama Study dataset did not distinguish between healthy controls and controls with non-AD forms of dementia, both of which are included in the non-AD groups that we report.

**Figure 5:**
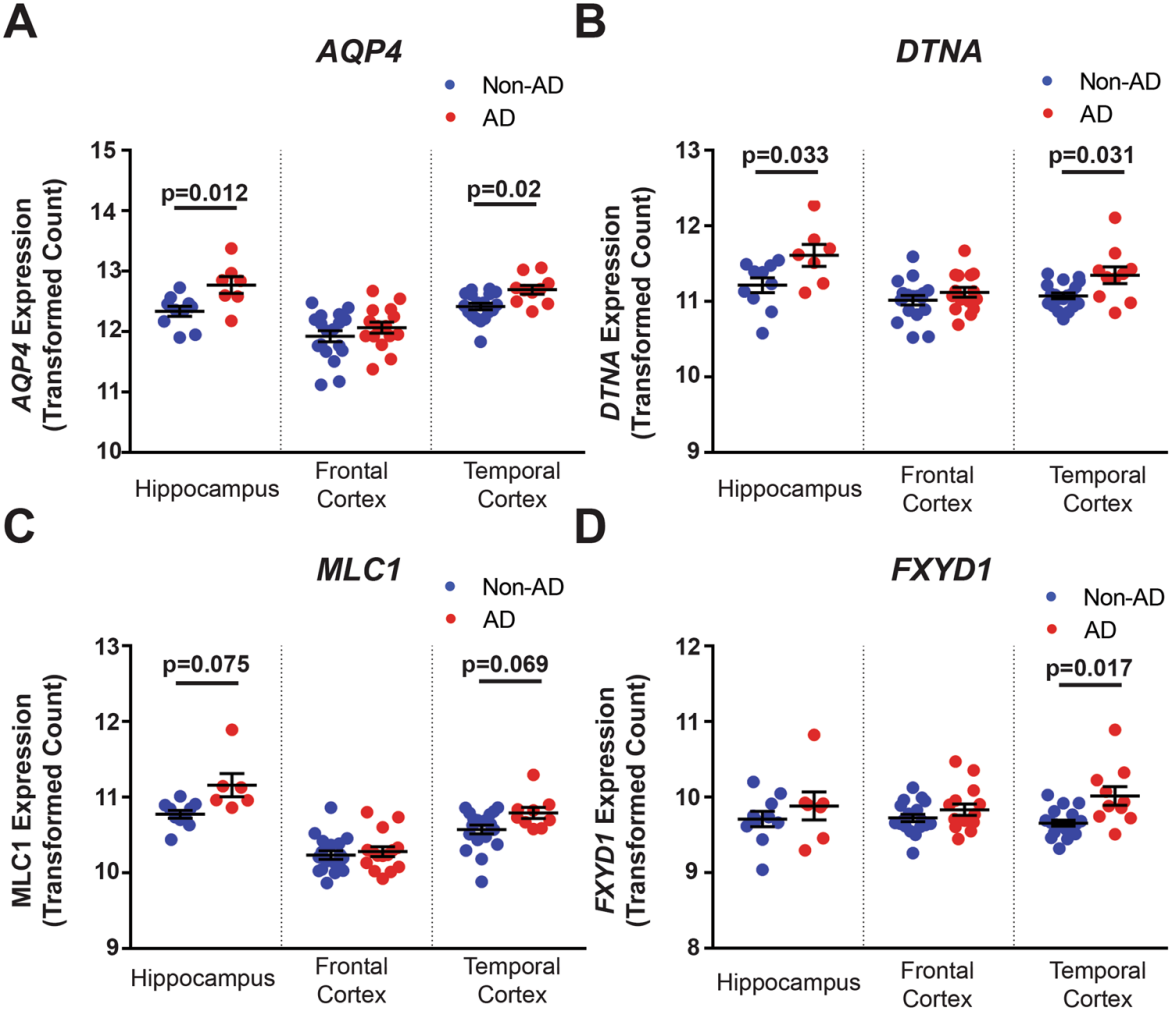
Validation of gene expression changes in the Hisayama microarray dataset. The association between gene expression and dementia status evaluated in an independently generated AD cohort for four of the DAC and DAC-associated genes identified: *AQP4*, *DTNA*, *MLC1*, and *FXYD1*. Expression changes measured in three regions: HIP, FCX and TCX. For each gene, plots describe associations with dementia status in each brain region (**A**–**D**). In HIP, elevated expression of *AQP4* (p = 0.012) and *DTNA* (p = 0.033) significantly predict dementia status (red, n = 7) compared to healthy individuals (blue, n = 10), while *MLC1* (p = 0.075) shows a trend towards elevated expression. In temporal cortex *AQP4* (p = 0.02), *DTNA* (p = 0.031) and *FXYD1* (p = 0.017) all significantly predict dementia status (red, n = 10) compared to healthy subjects (blue, n = 19) while *MLC1* (p = 0.069) again exhibits a trend towards increased expression. No differences were observed between dementia (red, n = 15) and non-dementia subjects (blue, n = 18). Statistical associations calculated with logistic regression. The bars in (**A**–**D**) represent the mean ± the standard error of the mean (S.E.M.). Complete statistical information found in Table 4.

Evaluation of this microarray data revealed strikingly consistent associations with the Allen Aging, Dementia, and TBI dataset for each of the four genes (*AQP4*, *DTNA*, *MLC1*, *FXYD1*; Fig. 4 and Table 4). In FCX, no significant associations are observed for any of the four genes. In HIP, both *AQP4* (Fig. 5A; z = 2.85, p = 0.012) and *DTNA* (Fig. 5B; z = 2.35, p = 0.033) show significantly elevated expression among AD subjects. Although *MLC1* expression was generally higher in AD compared to non-AD HIP, this effect was not significant (Fig. 5C; z = 1.78, p = 0.075). Hippocampal *FXYD1* expression was not significantly different between AD and non-AD subjects. In TCX *AQP4* (Fig. 5A; z = 2.32, p = 0.02), *DTNA* (Fig. 5B; z = 2.16, p = 0.031), and *FXYD1* (Fig. 5D; z = 2.39, p = 0.017) all show significant increases in expression in AD subjects, while *MLC1* demonstrates only a trend towards increased expression among AD subjects (Fig. 5C, z = 1.82, p = 0.069).

**Table 4:** Associations of gene expression and protein expression with Alzheimer’s diagnosis and pathology in independently generated AD cohorts.

Study	Region	Gene/Protein	Coefficient	z-stat	p-value
Gene Expression (Hisayama Study)	Frontal Cortex	*AQP4*	1.092	1.08	0.28
		*DTNA*	1.633	1.13	0.26
		*MLC1*	0.777	0.53	0.59
		*FXYD1*	1.905	1.23	0.22
	Temporal Cortex	*AQP4*	6.34	2.32	**0**.**02**
		*DTNA*	5.014	2.16	**0**.**031**
		*MLC1*	5.298	1.82	*0*.*069*
		*FXYD1*	6.058	2.39	**0**.**017**
	Hippocampus	*AQP4*	5.472	2.85	**0**.**012**
		*DTNA*	4.312	2.35	**0**.**033**
		*MLC1*	15.68	1.78	*0*.*075*
		*FXYD1*	1.249	0.88	0.377
Protein Expression (Oregon Brain Bank)	Frontal Cortex	AQP4	−1.535	−0.94	0.35
		DTNA	0.064	0.33	0.74
		MLC1	0.406	0.22	0.83
		FXYD1	0.981	0.87	0.38
	Hippocampus	AQP4	0.281	0.17	0.864
		DTNA	2.932	2.16	**0**.**031**
		MLC1	9.25	2.25	**0**.**025**
		FXYD1	1.961	1.66	*0*.*096*
Hippocampal Protein Expression vs. Pathology (Oregon Brain Bank)	Neuritic Plaques	AQP4	0.684	0.38	0.705
		DTNA	2.708	2.04	**0**.**042**
		MLC1	0.253	0.15	0.88
		FXYD1	1.815	1.8	*0*.*073*
	Braak Staging	AQP4	0.544	0.51	0.612
		DTNA	2.732	2.01	**0**.**048**
		MLC1	9.688	2.21	**0**.**027**
		FXYD1	1.143	1.54	0.12
	Cerebral Amyloid Angiopathy	AQP4	−1.939	−0.56	0.575
		DTNA	2.591	1.86	*0*.*063*
		MLC1	−1.478	−0.51	0.61
		FXYD1	0.885	0.81	0.42

Correlation coefficients, z-statistics and p-values for the uncorrected logistic regression between gene/protein expression and AD diagnosis (top two groups) or AD pathologies (bottom group). P < 0.05 labeled in bold. Italics indicates statistical trends (P < 0.1). Positive z statistics represent a positive association between gene expression and pathology or diagnosis, while negative statistics represent an inverse association.

**Figure 6:**
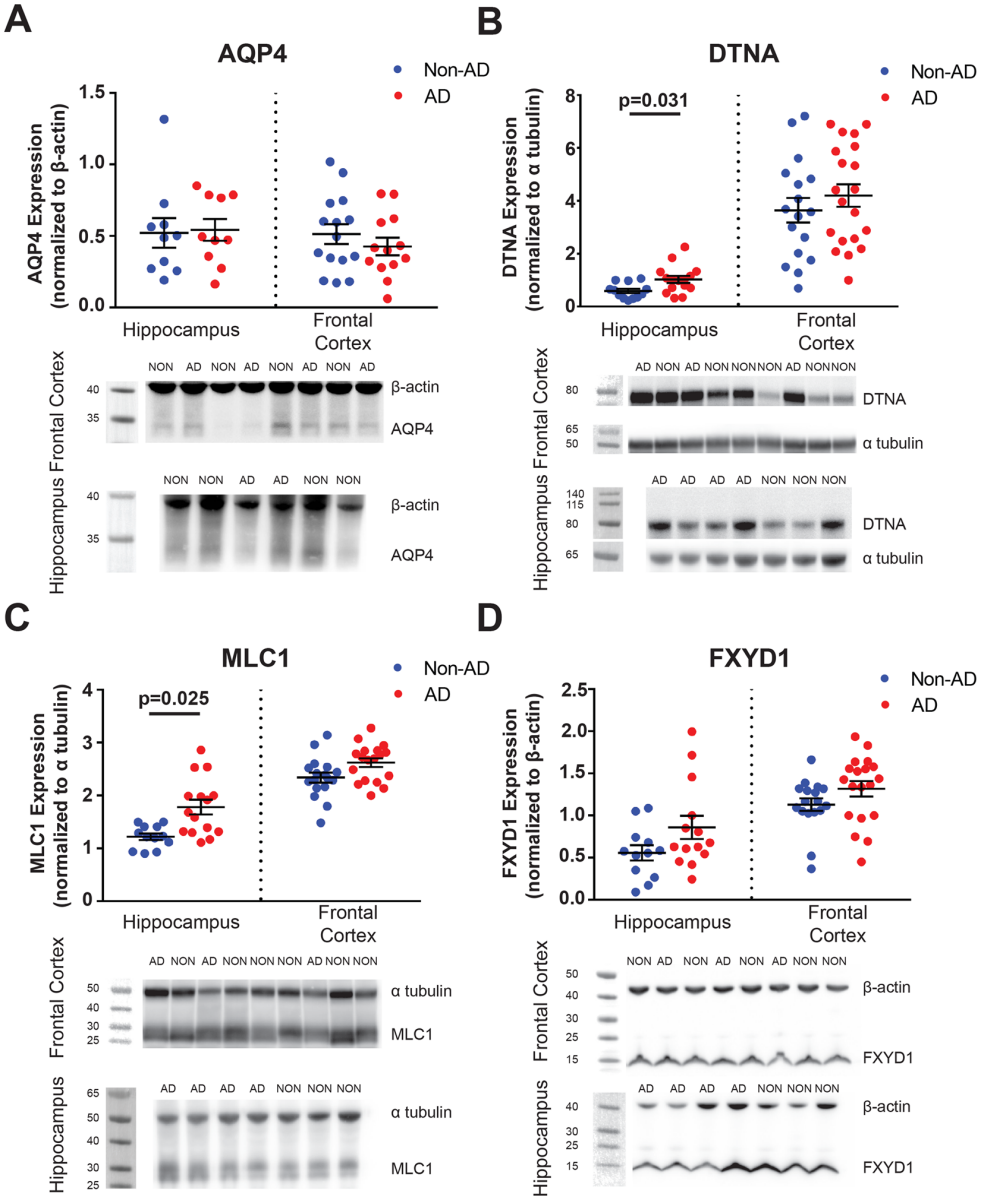
Assessment of the association between protein expression changes and Alzheimer’s disease status. Protein expression and associations with AD status for AQP4, DTNA, MLC1, and FXYD1. Expression changes measured in HIP (n_dementia_ = 10–15, n_non-dementia_ = 10–12) and FCX (n_dementia_ = 13–20, n_non-dementia_ = 15–17). For each gene, plots describe associations with AD status in each brain region (**A**–**D**). In HIP, elevated expression of DTNA (p = 0.031) and MLC1 (p = 0.025) significantly predict dementia status. Statistical associations calculated with logistic regression. The bars in (**A**–**D**) represent the mean ± the standard error of the mean (S.E.M.). Complete statistical information found in Table 4. Representative blots have been cropped for clarity. Dotted lines represent the boundary between cropped images. All blots were processed in parallel across subjects. Full length representative blots can be found in Supplementary Fig. 2.

To determine if gene expression changes are indicative of protein expression levels, we evaluated AQP4, DTNA, MLC1 and FXYD1 expression by Western blot in fresh-frozen grey matter tissue samples available through the Oregon Brain Bank (Supplementary Fig. 2). Samples were obtained from two brain regions: HIP and FCX. Subjects were selected based on the absence of clino-pathological diagnosis of vascular or mixed dementia, hippocampal sclerosis, Lewy body dementia, Parkinson’s disease, tumor, or other non-AD pathologies, to best define the relationship between expression of these proteins and AD specifically. The resulting case series consisted of 35 FCX samples, including 15 from non-AD subjects and 20 from AD subjects; and 27 HIP samples, including 12 from non-AD subjects and 15 from AD subjects. Subject gender was roughly even within each group (43% male among non-AD; 46% male among AD subjects). As in the Hisayama dataset, there was a significant difference in the age distribution between AD subjects (median = 84.9 years, IQR = 10.0 years) and Non-AD subjects (median = 96 years, IQR = 5.6 years), which is likely attributable to the exclusion of subjects with vascular or mixed dementia. No associations between protein expression and dementia status were observed in FCX for the four genes tested (Fig. 6 and Table 4). Within HIP, significantly increased expression levels were observed in AD subjects for both DTNA (Fig. 6B, z = 2.16, p = 0.031) and MLC1 (Fig. 6C, z = 2.25, p = 0.025), while only a trend was observed towards increased FXYD1 expression subjects with AD diagnosis (Fig. 6D, z = 1.66, p = 0.096). AQP4 did not demonstrate any significant association with AD diagnosis (Fig. 6A).

Next, we assessed the relationship between protein-level expression changes, specifically in the HIP region, and pathological indicators of protein aggregation: Braak stage, neuritic plaque burden, and cerebral amyloid angiopathy. Pathological neuritic plaque burden was significantly associated with elevated hippocampal DTNA expression (Table 4, z = 2.04, p = 0.042). Both hippocampal DTNA and MLC1 expression were significantly associated with Braak stage (Table 4; z = 2.01, p = 0.048; z = 2.21, p = 0.027; respectively). These additional gene and protein expression datasets confirm that changes in the expression of *AQP4*, DAC and candidate endfoot genes and their encoded proteins both predict dementia status and are associated with Alzheimer’s pathology, in particular cortical tau pathology.

## Discussion

In the present study, we utilized publicly available transcriptomic datasets to generate an unbiased, large-scale assessment of gene expression to evaluate potential associations between expression of perivascular astroglial endfoot components, dementia status and AD pathology in human brain tissue. This approach revealed that variation in several known genes encoding structural elements of the astroglial endfoot domain are associated with dementia status. Unexpectedly, differences in expression of these genes was also associated with P-tau pathology in TCX. WGCNA-based clustering approaches revealed several genes that may represent novel elements of the astrocytic endfoot domain. These candidate genes also exhibit associations with TCX P-tau pathology, and in many cases, hippocampal expression of these genes predicted dementia status. Finally, we validated these findings at the transcript and protein level in two independent AD cohorts, demonstrating that expression of the DAC protein DTNA (dystrobrevin) and the candidate endfoot protein MLC1 (megalencepalic leukoencephalopathy with subcortical cysts 1) predicts AD status and are associated with cortical tau pathology.

The perivascular astroglial water channel AQP4 supports perivascular CSF-interstitial fluid exchange, as deletion of the *Aqp4* gene in mice slows CSF influx into the brain parenchyma and the clearance of solutes, including Aβ, from the brain parenchyma^8^. Impairment of glymphatic function in the aging and post-traumatic rodent brain is associated with increased AQP4 expression and loss of perivascular AQP4 localization^10,11,19^. In human post-mortem tissue, increasing AQP4 expression and loss of perivascular localization is associated with AD status, Aβ plaque burden and NFT pathology^14^. Yet the cellular and molecular underpinnings of glymphatic pathway function beyond AQP4 have not yet been defined. Beyond the glymphatic system, the astrocytic endfoot contributes to physiological processes such as fluid homeostasis, neurovascular coupling and brain metabolism. Under pathological conditions it also plays a critical role the formation and resolution to cerebral edema in response to brain injury^20^. Identification of novel elements of the perivascular endfoot domain that contribute to these processes may provide new insight into the pathogenesis and potential treatment of neurodegenerative or neurovascular disorders. The present observation that increased *AQP4* expression was associated with increased Aβ levels in the parietal cortex is consistent with prior studies of glymphatic function demonstrating a role of AQP4 in Aβ clearance and deposition^8,9,11^. Similarly, the observed association between increasing *AQP4* expression in the HIP and dementia status is consistent with our prior finding that increased AQP4 expression and loss of perivascular localization predicted AD status, and that naturally occurring single-nucleotide polymorphisms in the human *AQP4* gene are associated with altered rates of neurocognitive decline among subjects with AD^14,21^.

Transcriptomic analysis of *AQP4* and the perivascular astroglial DAC genes *SNTA1* (α-syntrophin), *DTNA* (dystrobrevin), *DAG1* (dystroglycan) and *DMD* (dystrophin) across both the human developing brain^18^ and in the aging brain (present study) showed that *AQP4*, *DTNA* and *SNTA1* exhibit a common expression profile that is shared by a group of astroglial genes. These datasets showed striking overlap in expression profiles with nine novel candidates identified in the endfoot-enriched cluster in both datasets. Furthermore, about one third (13 of 41 in the developmental dataset, 3 of 11 in the present dataset) of these endfoot candidate gene products have previously been localized to the perivascular endfoot domain^22–24^. For example, *MLC1* encodes a putative astroglial membrane transporter of unknown function that associates with AQP4 and other DAC proteins in the perivascular astrocytic endfoot^25^. In contrast, the distribution and function of many of the remaining gene products remains unknown. The clustering profile of *AQP4*, the DAC genes (excluding *DMD*), and these endfoot candidates is largely distinct from that of other common astrocytic genes such as *GFAP*, *S100β* and *ALDH1L1*, suggesting that these may represent a cluster of genes that contribute to perivascular astroglial function. Initial assessment of functions within this group by gene ontology analysis reveals enrichment for molecular functions including amino acid binding and transmembrane receptor protein kinase activity (data not shown).

Taking advantage of RNAseq datasets allowed for an unbiased screen for novel gene candidates, but importantly does not provide information on protein-level expression. Western blot data from an independent AD cohort suggests that at least for the subset of the gene products evaluated, the associations between gene expression, AD status and neurofibrillary pathology were faithfully recapitulated at the protein expression level. Furthermore, these results exhibit the same regional specificity, as expression of DTNA and MLC1 within HIP, but not the FCX, predicted AD status. It is important to note that this validation cohort was relatively small and did not include TCX tissue, the region exhibiting the most robust associations between P-tau pathology and gene expression data in the Allen Aging, Dementia and TBI study. Thus, a more complete assessment of protein level expression as well as histological evaluation of localization patterns for the identified genes is warranted. Localization data obtained for novel candidates will be of particular interest due to the established loss of AQP4 localization in pathological states without robust changes in overall expression.

The surprisingly strong associations between TCX DAC gene expression and local P-tau levels observed in all three datasets suggests that astroglial endfoot function may play a previously unrecognized role in the dynamics of NFT formation. This notion is supported by the observed association between changes in AQP4 expression and localization and Braak stage in human post-mortem tissue^14^ and by the increased P-tau levels observed in *Aqp4*^−/−^ mice after experimental TBI^10^. The functional link between the astroglial endfoot, which has been associated with extracellular solute homeostasis, and the development of intracellular aggregates of tau, remains speculative. One possibility is that perivascular endfoot function may influence the inter-cellular spread of tau aggregates. The neuroanatomical progression of tau pathology through the course of AD reflected in the Braak staging system is now widely believed to result from the prion-like spread of misfolded tau between neighboring cells within neural networks^6^. Such prion-like spread has been observed for several protein aggregates related to neurodegenerative disease, including tau and α–synuclein^26^. In an experimental setting, tau aggregates isolated from human brains can seed NFT formation in the wild type mouse brain, which does not form native NFTs, leading to the propagation of NFTs through neuroanatomically connected brain regions^27^. Implicit within this model, is that intracellular tau aggregates must be released into and taken up out of the extracellular compartment. Cellular release and uptake of tau aggregates has been observed both *in vitro* and *in vivo*, where release of monomeric tau into the brain interstitium in response to excitatory synaptic activity has been detected by microdialysis^7,28,29^. By altering the uptake and clearance dynamics of tau or tau aggregates within the extracellular compartment, changes in perivascular endfoot function may influence pathological tau propagation.

Among the three datasets, a consistent association between gene expression in temporal cortex and P-tau levels was observed, but not with dementia status. One possible explanation is that these differences reflect the regional progression of AD pathology, specifically neurofibrillary tau tangle formation, with regard to clinical severity. TCX P-tau burden is associated with late-preclinical stages of AD while tau pathology is not seen in HIP until the latest stages of AD, when it manifests clinically. If changes in DAC gene expression represent a response to tau phosphorylation, these changes may occur in TCX prior to the HIP. The lack of associations in PCX and FCX may reflect the cellular properties of the region that render it highly vulnerability to tau formation. In contrast, hippocampal gene expression and clino-pathological dementia status are associated, but without consistent associations to Aβ or tau pathology. A second possibility is that in HIP, these changes in expression do not occur until late stages of disease progression when clinical diagnosis is a stronger indicator of disease progression than tau burden. Further investigation of the timing of glial responses to disease progression are needed to better resolve the source of these regional differences.

One caveat to the present study is the inability to distinguish astrocytic gene expression from expression in other cell types in the datasets utilized. Though we controlled for this to the best of our ability by cross-referencing with single-cell transcriptomic data, heterogeneity among astrocyte populations remains a major gap in our understanding of the associations between different astroglial genes and local pathological features, such as P-tau levels. It remains an open question as to the specific cell types or subpopulations in which the novel ‘endfoot candidate’ genes are expressed in. Other factors including neuronal subtypes and density, vascular supply and blood-brain barrier function, as well as proximity to white matter, subarachnoid or ventricular CSF compartments, also likely contribute to the variability in associations between regional pathology and gene expression. This further supports the need for validation of the present study in a manner that assesses cell-type expression and localization.

The present study demonstrated a robust association between both established and candidate molecular components of the perivascular astrocytic endfoot and dementia status and markers of AD pathology. Importantly, these novel astroglial gene candidates were identified through a non-biased clustering approach based on two distinct human transcriptomic datasets and exhibited a striking and near-universal association with P-tau levels within the TCX, albeit all associations were identified using correlation-based approaches. While we propose these candidate endfoot components exert these effects via their putative role in glymphatic pathway function, this remains to be experimentally validated. The strong association with both dementia status and P-tau pathology in a large cohort of human subjects indicates that perivascular astrocytic endfoot function, and specifically the 15 gene products (*AQP4*, the DAC genes, and the 11 candidate endfoot genes) may represent novel regulators of tau aggregation and the propagation of tau pathology in the setting of AD.

## Methods

### Data Sources

Links and information regarding the databases used in this study are available in Supplementary Table 1.

### Allen Brain Institute Aging, Dementia and TBI Database

This publicly available dataset contains RNAseq–derived transcriptome data from 107 individuals aged 77 and older obtained from the Adult Change in Thought (ACT) cohort^30^. RNAseq data were collected from up to 4 brain regions within each subject, with over 20,000 genes read from each region, totaling 377 independent samples. Detailed description of tissue collection and processing is available in the database documentation (http://help.brain-map.org/display/aging/Documentation). Entrez IDs (as provided by Allen Brain Institute) were used for protein identification and subsequent analysis.

### Hisayama Study Gene Array Dataset

The microarray data from the Hisayama study is publicly available through the Pubmed GEO database (GDS4758) and includes HIP, FCX, and TCX data from 79 subjects ranging in age from 54 to 105 years of age. Details regarding subjects and sample collection is found on the “sample subsets” page of the database (https://www.ncbi.nlm.nih.gov/sites/GDSbrowser?acc=GDS4758#details). Inclusion in the AD group was based upon histopathological evaluation based on the Consortium to Establish a Registry for Alzheimer’s Disease (CERAD) guidelines^31^ and Braak staging^32,33^. Importantly, this study did not distinguish between healthy control subjects and control subjects with vascular dementia or other mixed dementias. This is different than the control group distinction made in the Oregon Brain Bank Dataset (described below).

### Oregon Brain Bank Dataset

Frontal cortex grey matter or hippocampal samples were obtained from 43 subjects in the Oregon Health & Science University Layton Aging and Alzheimer’s Disease Center and associated postmortem tissue repository, or the Oregon Brain Bank. Volunteers signed written informed consent. All aged participants were community-dwelling individuals with no known neurological disease or with a clinical history of AD as established by neurologic evaluation in the Layton Aging and Alzheimer Disease Center in accordance with established consensus criteria. Brain autopsy was performed on all participants after consent was obtained from the next of kin and in accordance with Oregon Health and Science University guidelines (IRB 00001623). Brains in the Oregon Brain Bank underwent neuropathological evaluation for Aβ plaque density, neurofibrillary pathology, and vascular pathology based on CERAD guidelines^31^ and Braak staging^33^. The cohort utilized in this study was selected to minimize inclusion of individuals with secondary pathologies beyond clino-pathological diagnosis of AD, including vascular or mixed dementia, Lewy body dementia, and hippocampal sclerosis. Consequently the ‘Non-AD’ group includes both cognitively intact subjects and those with a clinical diagnosis of mild cognitive impairment.

### Statistical analysis

All statistical analyses were performed using R 3.2 and 3.3^34^.

#### Allen Aging, Dementia and TBI dataset

Logistic regression was utilized to assess the relationship between propensity for a diagnosis of dementia at death, formatted as a dichotomous factor, against mRNA expression of the genes of interest. Mann-Whitney *U* test was used to assess group-wise differences in demographic information. When evaluating the association of gene expression to Alzheimer’s pathology, ordinary least-squares (OLS) regression was used to model the CNS pathology levels as continuous outcomes. Model diagnostics revealed the pathology measures to exhibit a right-skew and inconsistent variance of the residuals, therefore logarithmic transforms were applied to the outcomes as necessary. All regression models controlled for age, APOε4 carrier status and a previous history of TBI. Age was framed as a continuous factor although set in 5-year increments across the dataset to account for reporting of age ranges over 95 years of age. APOε4 carrier status was a dichotomous variable to indicate the subject as a heterozygous or homozygous minor allele carrier and TBI was set as a dichotomous variable based on a reported history of brain injury. Gene expression was first correlated with pathology across the entire cohort with subsequent analysis directly contrasting the correlation based on dementia status by assessing the interaction between RNAseq and clinical dementia diagnosis.

Model fit and integrity were examined using a combination of formal fit criteria, including Cook’s distance and the standardized difference of the betas, and visual inspection of the residual plots. Results were considered significant at p < 0.05 after repeated comparison correction to account for the multiple response variables. Test statistics were set *a priori* to establish power at 80% with an α value of 0.05. False discovery correction was first used across the entire cohort of assessed models to allow for only 5% of erroneous discoveries in the significant results. The significance of this selection set was then further adjusted using the more stringent Holm-Sidak family-wise error rate correction to correct for multiple comparisons with adjustments carried out separately by individual brain regions due to their inherent differences in biology. These adjusted P values were used to determine final model significance.

#### Hisayama gene array and Oregon Brain Bank datasets

Logistic regression was used to assess the relationship between a diagnosis of dementia at death against mRNA expression of the genes of interest. Logistic regression was again used to model the relationship between AD diagnosis and CNS pathology levels as the ranges are too truncated to be used as linearly continuous outcomes. “Pathological” states were defined as follows; Braak Stage ≥5, Neuritic Plaques ≥3, Cerebral amyloid angiopathy ≥3. In neither of these datasets were corrections made to account for the covariates. This was due to two features of these datasets. First, both datasets were chosen as validation cohorts and therefore have small sample sizes. Compared to the large discovery cohort, the small sample sizes in these validation datasets made correction for covariates impracticable. Second, both validation datasets were selected because they originated in AD-focused cohorts, allowing a more discreet assessment of associations between the expression of four discrete genes and their products with AD diagnoses or pathological indicators. Consequently, this resulted in substantial age gaps between AD and non-AD cohorts due to the well-established age dependency of the disease.

In contrast to the exploratory nature of the correlation network analysis, the validation cohorts were chosen to explicitly confirm the effects of four heavily implicated genes in particular: the known endfoot elements AQP4 and DTNA, and the WGCNA-identified candidates MLC1 and FXYD1. Furthermore, gene effects were compared within brain regions that had been observed to be biologically distinct with respect to pathology and RNAseq levels. Based on these a priori notions, multiple comparison adjustments were not performed with original p-values presented. Although this does lead to a reduction in the post-hoc power of these validation datasets, it is also able to highlight the sheer number of observed significant relationships across a variety of multiple experimental studies and frameworks.

### Weighted Gene Correlation Network Analysis (WGCNA)

Weighted Gene Correlation Network Analysis (WGCNA) was implemented using the WGCNA package in R (http://www.genetics.ucla.edu/labs/horvath/CoexpressionNetwork/, RRID: SCR_003302)^35–37^. Four independent networks were generated from the dataset: single networks were constructed within the full, dementia-only, and non-dementia-only populations and a consensus network was generated across dementia status. Initial filtering was applied prior to each analysis to eliminate spurious genes that had fragments per kilobase per million (FPKM) values of less than 10 in 90% or more of total reads resulting in approximately 7,000 genes per brain region. Adjacency matrices for subjects with dementia and dementia-free populations were generated by calculating biweight midcorrelations for gene pairs. Soft thresholding was used to establish a connection weight between each gene pair within each brain region^37^. Threshold values were between 4–7 for all analyses and were determined by manual assessment of scale-free topology after plotting. Next, a topographical overlap matrix (TOM) was generated to determine adjacency for gene pairs. Component-wise minimum of the single network TOMs were used to generate the dementia: non-dementia consensus TOM. Dissimilarity matrices were generated by subtracting the TOM values from 1. Using these values, hierarchical clustering defined modules based on highly interconnected expression profiles. In addition to TOM values, the parameters used from clustering included a minimum module size of 30 and an unsigned network. Eigengenes were calculated for each module to allow for evaluation of module expression with pathology data and hub gene identification. Hub genes were determined by calculating the correlation between each gene in a module and each modules calculated eigengene.

### Cluster-based analysis

The gene modules in which DAC genes were most highly expressed were extracted from each brain region’s WGCNA-based clusters. For this analysis, the clusters generated by the full, single network WGCNA was utilized as it resulted in smaller cluster sizes, and generally greater intramodular connectivity for DAC genes than in the consensus network (Supplementary Table 4). Genes with robust astrocytic expression were extracted by thresholding out genes with less than 50% of total expression derived from astrocytes. Cell-type expression values were derived from a separate cell-type RNAseq transcriptome database, resulting in 50 to 100 astrocytic genes per cluster^17^. Pearson correlation coefficient was used to identify genes highly coexpressed with DAC complex genes across subjects. A gene was only considered as coexpressing with DAC components after a conservative correlation of r > 0.65 (p < 1e25) was observed with at least two of the DAC genes. These conservative thresholds on Pearson’s correlation coefficient were selected according to the most stringent Bonferonni-based correction possible under this framework (50281 transcripts contrasted against 4 DAC members across 3 brain regions) and to indicate effect sizes that have traditionally been cited as notably powerful (Pearson’s r > 0.6 and Cohen’s d >1.5). Genes were only selected that demonstrated significant correlation to AQP4 or the DAC genes in at least 2 of the 3 brain regions.

### Western Blot

Human frontal cortex grey matter or hippocampus frozen tissue was homogenized by sonication in tissue homogenization buffer (62.5 mM Tris (pH 6.8), 10% glycerol, and 2% SDS) on ice. BCA assays were performed to determine the protein concentration (ThermoFisher Cat# 23225). Additional 0.1% bromophenol blue and 50 mM DTT were added to the sample after the BCA reaction and before the denaturation at 95 °C for 5 minutes. Depending on the sensitivity of the antibodies, 50–100 µg of samples were loaded for electrophoresis. Gels were transferred using BioRad Trans-Blot® Turbo™ Transfer System and blots were detected using BioRad ChemiDoc™ Touch Imaging System. Primary antibody used in this study are: AQP4 (1:500, Millipore, Cat# AB3594), FXYD1 (1:500, Abcam, Cat# ab76597), MLC1 (1:1000, Abcam, Cat# ab186436) and DTNA (1:500, BD, Cat# BDB610766). Secondary antibodies were purchased from GE healthcare (1:1000). Band intensity was quantified using ImageJ. The ratio in chemiluminescent signal of the target band relative to the loading control band was measured and normalized to the reference sample on each gel. The average value of two batches were used for quantification.

## Electronic supplementary material

Supplementary Information

Supplementary Dataset 1

## References

[1] Serrano-Pozo A, Frosch MP, Masliah E, Hyman BT (2011). Neuropathological alterations in Alzheimer disease. Cold Spring Harb Perspect Med.

[2] Cirrito JR (2008). Endocytosis is required for synaptic activity-dependent release of amyloid-beta *in vivo*. Neuron.

[3] Mawuenyega KG (2010). Decreased clearance of CNS beta-amyloid in Alzheimer’s disease. Science (New York, N.Y.).

[4] Patterson BW (2015). Age and amyloid effects on human central nervous system amyloid-beta kinetics. Annals of neurology.

[5] Guo JL, Lee VM (2011). Seeding of normal Tau by pathological Tau conformers drives pathogenesis of Alzheimer-like tangles. J Biol Chem.

[6] Liu L (2012). Trans-synaptic spread of tau pathology *in vivo*. Plos One.

[7] Yamada K (2014). Neuronal activity regulates extracellular tau *in vivo*. J Exp Med.

[8] Iliff JJ (2012). A paravascular pathway facilitates CSF flow through the brain parenchyma and the clearance of interstitial solutes, including amyloid beta. Sci Transl Med.

[9] Xu Z (2015). Deletion of aquaporin-4 in APP/PS1 mice exacerbates brain Abeta accumulation and memory deficits. Mol Neurodegener.

[10] Iliff JJ (2014). Impairment of glymphatic pathway function promotes tau pathology after traumatic brain injury. J Neurosci.

[11] Kress BT (2014). Impairment of paravascular clearance pathways in the aging brain. Ann Neurol.

[12] Eide PK, Ringstad G (2015). MRI with intrathecal MRI gadolinium contrast medium administration: a possible method to assess glymphatic function in human brain. Acta Radiol Open.

[13] Ringstad G, Vatnehol SAS, Eide PK (2017). Glymphatic MRI in idiopathic normal pressure hydrocephalus. Brain: a journal of neurology.

[14] Zeppenfeld DM (2017). Association of Perivascular Localization of Aquaporin-4 With Cognition and Alzheimer Disease in Aging Brains. JAMA neurology.

[15] Allen Institute for Brain Science. Aging, Dementia and TBI. Available from, http://aging.brain-map.org/ (2016).

[16] Allen Institute for Brain Science. Allen Cell Types Database. Available from, http://celltypes.brain-map.org/ (2015).

[17] Zhang Y (2016). Purification and Characterization of Progenitor and Mature Human Astrocytes Reveals Transcriptional and Functional Differences with Mouse. Neuron.

[18] Simon, M. J., Murchison, C. & Iliff, J. J. A transcriptome-based assessment of the astrocytic dystrophin-associated complex in the developing human brain. *Journal of neuroscience research*, 10.1002/jnr.24082 (2017). 10.1002/jnr.24082 PMC5995340 28509351

[19] Zhao ZA (2017). Perivascular AQP4 dysregulation in the hippocampal CA1 area after traumatic brain injury is alleviated by adenosine A2A receptor inactivation. Sci Rep.

[20] Nagelhus EA, Ottersen OP (2013). Physiological roles of aquaporin-4 in brain. Physiol Rev.

[21] Burfeind, K. G. *et al*. The effects of noncoding aquaporin-4 single-nucleotide polymorphisms on cognition and functional progression of Alzheimer’s disease. *Alzheimer’s & Dementia: Translational Research & Clinical Interventions* **3**, 348–359, 10.1016/j.trci.2017.05.001. 10.1016/j.trci.2017.05.001 PMC5651426 29067342

[22] Langer J (2017). Rapid sodium signaling couples glutamate uptake to breakdown of ATP in perivascular astrocyte endfeet. Glia.

[23] Schreiner AE (2014). Laminar and subcellular heterogeneity of GLAST and GLT-1 immunoreactivity in the developing postnatal mouse hippocampus. The Journal of comparative neurology.

[24] Flugge G, Araya-Callis C, Garea-Rodriguez E, Stadelmann-Nessler C, Fuchs E (2014). NDRG2 as a marker protein for brain astrocytes. Cell and tissue research.

[25] Boor I (2007). MLC1 is associated with the dystrophin-glycoprotein complex at astrocytic endfeet. Acta Neuropathol.

[26] Guo JL, Lee VM (2014). Cell-to-cell transmission of pathogenic proteins in neurodegenerative diseases. Nat Med.

[27] Recasens A (2014). Lewy body extracts from Parkinson disease brains trigger alpha-synuclein pathology and neurodegeneration in mice and monkeys. Annals of neurology.

[28] Frost B, Jacks RL, Diamond MI (2009). Propagation of tau misfolding from the outside to the inside of a cell. J Biol Chem.

[29] Pooler AM, Phillips EC, Lau DH, Noble W, Hanger DP (2013). Physiological release of endogenous tau is stimulated by neuronal activity. EMBO Rep.

[30] Miller, J. A. *et al*. Neuropathological and transcriptomic characteristics of the aged brain. *Elife* **6**, 10.7554/eLife.31126 (2017). 10.7554/eLife.31126 PMC5679757 29120328

[31] Mirra SS (1991). The Consortium to Establish a Registry for Alzheimer’s Disease (CERAD). Part II. Standardization of the neuropathologic assessment of Alzheimer’s disease. Neurology.

[32] Hokama M (2014). Altered expression of diabetes-related genes in Alzheimer’s disease brains: the Hisayama study. Cereb Cortex.

[33] Braak, H. & Braak, E. Staging of Alzheimer’s disease-related neurofibrillary changes. *Neurobiol Aging* **16**, 271–278; discussion 278–284 (1995). 10.1016/0197-4580(95)00021-6 7566337

[34] Team, R. C. R: A Language and Environment for Statistical Computing (2014).

[35] Langfelder P, Horvath S (2008). WGCNA: an R package for weighted correlation network analysis. BMC bioinformatics.

[36] Langfelder, P. & Horvath, S. Fast R Functions for Robust Correlations and Hierarchical Clustering. *J Stat Softw* **46** (2012). PMC3465711 23050260

[37] Zhang, B. & Horvath, S. A general framework for weighted gene co-expression network analysis. *Statistical applications in genetics and molecular biology* **4**, Article 17, 10.2202/1544-6115.1128 (2005). 10.2202/1544-6115.1128 16646834

