# Supplementary material for: Transcriptional network analysis of human astrocytic endfoot genes reveals region-specific associations with dementia status and tau pathology
Source: Sci Rep. 2018 Aug 17;8:12389. doi: 10.1038/s41598-018-30779-x (PMC6098119; doi:10.1038/s41598-018-30779-x)
Supplement: Supplementary file 1 — Supplementary Information [file 41598_2018_30779_MOESM1_ESM.pdf]

Supplementary Information

Transcriptional network analysis of human astrocytic endfoot genes reveals region-specific associations with dementia status and tau pathology

Matthew J. Simon[1,2], Marie X. Wang[1], Charles F. Murchison[3], Natalie E. Roese[1], Erin L. Boespflug[3], Randall L. Woltjer[4], Jeffrey J. Iliff[1,5] *

[1]Department of Anesthesiology and Perioperative Medicine, [2]Neuroscience Graduate Program, [3]Department of Neurology, [4]Department of Pathology, [5]Knight Cardiovascular Institute; Oregon Health & Science University, Portland, OR, USA.

***Corresponding Author:** Jeffrey J. Iliff, PhD

Department of Anesthesiology and Perioperative Medicine

Oregon Health & Science University

3181 SW Sam Jackson Park Rd.

Mail Code L459

Portland, OR 97239 USA

E-mail: iliffj@ohsu.edu

Phone: (503) 494-4047

Supplementary Figures

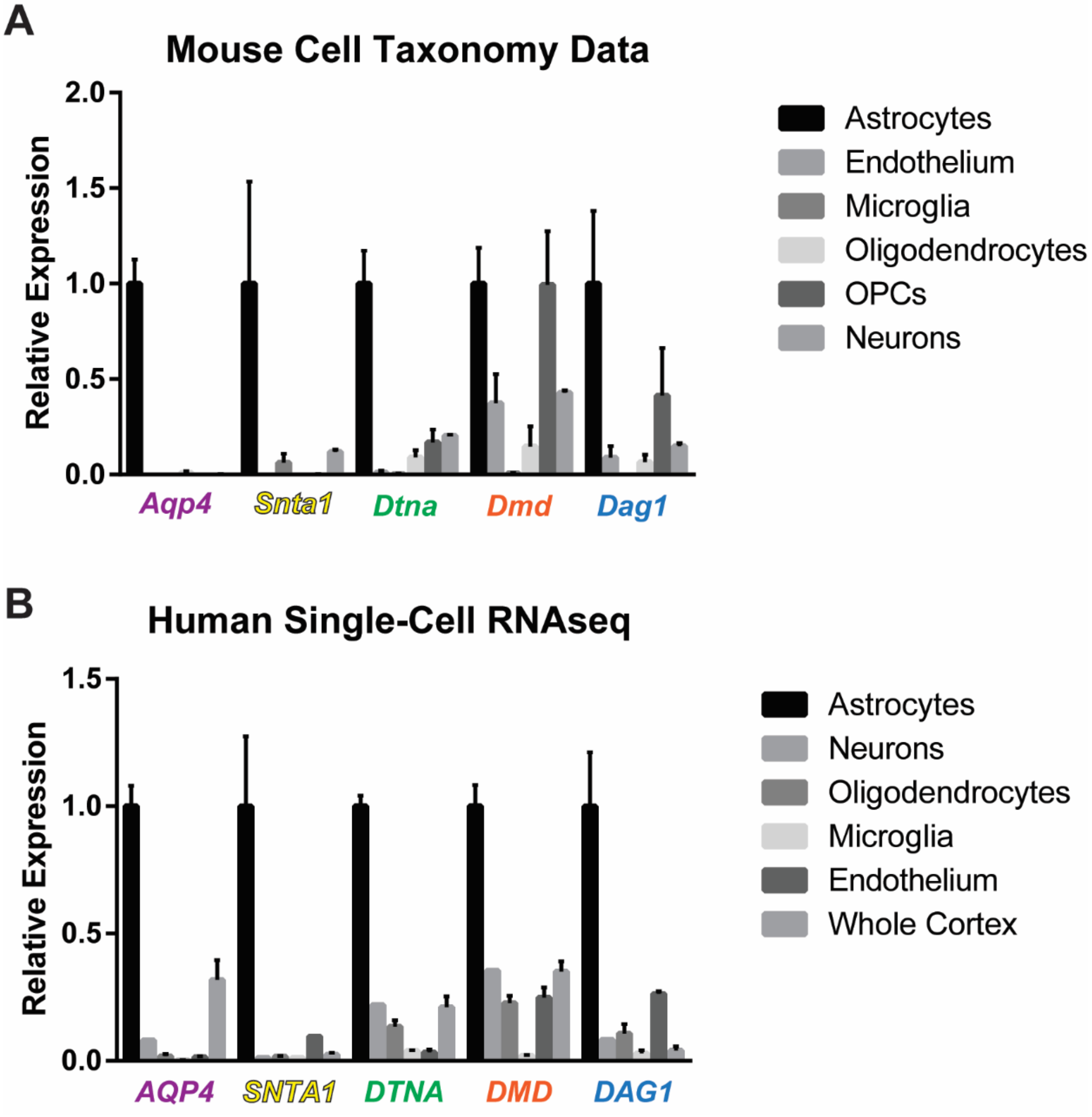

Supplementary Figure 1. AQP4 and the DAC genes are predominantly expressed in astrocytes. (A) RNA sequencing of single cells from adult mice demonstrate greater levels of expression of *Aqp4* and the other DAC genes in astrocytes relative to other measured cell types. Mouse RNA sequencing data was obtained from the Allen Cell Types Database. **(B)** Similarly, RNA sequencing data from human subjects again reveals significant enrichment for *Aqp4* and the DAC genes in astrocytes compared to other brain cell types. Human RNA sequencing data was obtained from the Brain RNAseq Database.

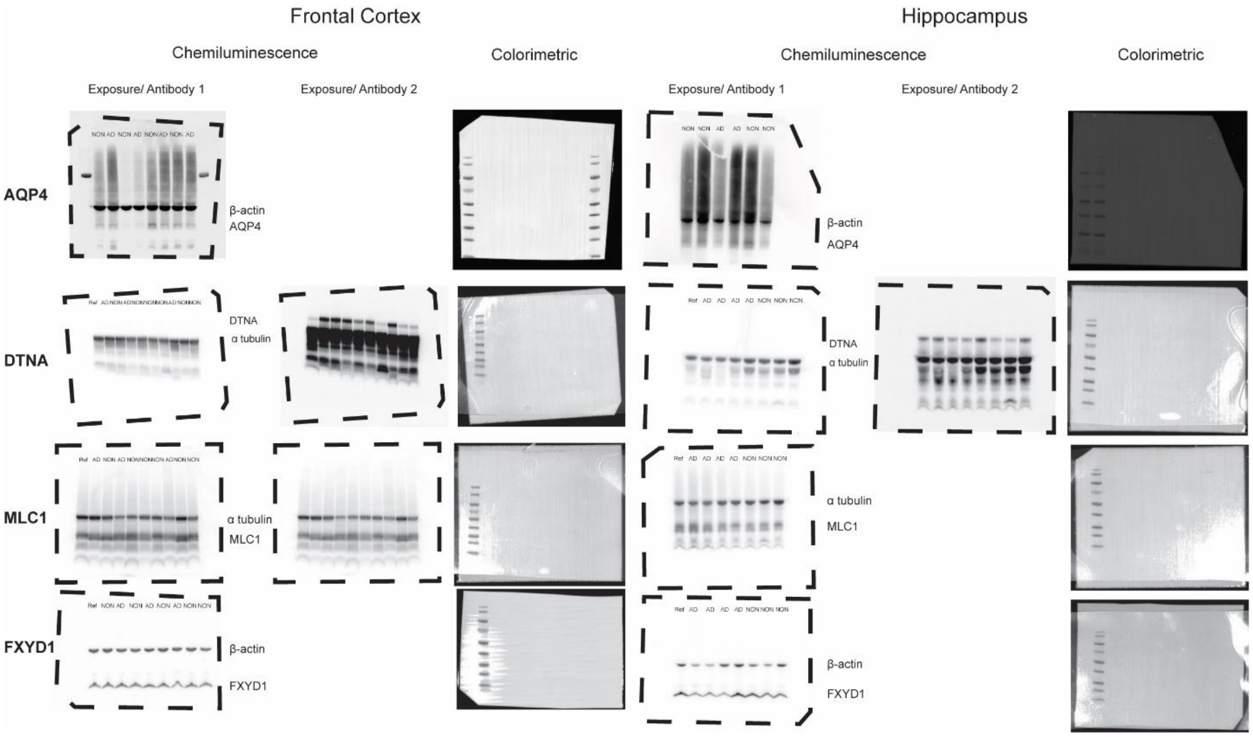

Supplementary Figure 2. Full length images of western blots for analysis of protein expression changes. The chemiluminescence and colorimetric images that were used for analysis in Figure 6 are provided. If multiple exposures or antibodies were used, both blots are included. Dotted lines represent the boundary of the blow when contrast is too high for distinction. Samples are labeled by AD diagnosis. AD= subject with AD, NON= non-demented subject, Ref = reference lane for normalization across blots.

Supplementary Tables

	Year	URL	Citation
Aging, Dementia and TBI	2016	http://aging.brain-map.org/	Miller et al. (2017)
Allen Cell Types Database	2015	http://celltypes.brain-map.org/	N/A
Brain RNA-Seq Database	2014	http://web.stanford.edu/group/barres_lab/brainseqMariko/brainseq2.html	Zhang, Y., et al. (2016).
Allen Cellular Taxonomy of the Mouse Visual Cortex Case Study	2016	http://casestudies.brain-map.org/celltax#section_explorea	Tasic, B., et al. (2016).
Postmortem Alzheimer's disease brains: Hisayama study	2014	https://www.ncbi.nlm.nih.gov/sites/GDSbrowser?acc=GDS4758	Hokama et al. (2014)

Supplementary Table 1: Publicly available databases used in this study. Descriptive information regarding each of the publicly available datasets utilized in this study, including relevant citations and URL link to the resource.

	Aging, Dementia & TBI dataset	Developing Human dataset (*Simon et al. 2017*)	Previously established at endfoot?
ACSS1	X	X	**No**
AMOT	X	X	**No**
BMPR1B	X	-	**No**
FGFR3	X	-	**No**
FXYD1	X	X	**No**
GLUD1	X	X	**No**
MLC1	X	X	**Yes** (*Boor et al. 2007*)
NDRG2	X	X	**Yes** (*Flugge et al. 2014*)
PBXIP1	X	X	**No**
PPAP2B	X	X	**No**
SLC1A3	X	X	**Yes** (*Langer et al. 2016; Schreiner et al. 2014*)

Supplementary Table 2: Established roles of WGCNA identified genes at the astrocytic endfoot.

A profile of the known associations between the DAC candidate genes and astrocytic endfoot localization. The second column illustrates the overlap between the cluster identified within the present Aging, Dementia & TBI dataset, and in a second dataset of developing human also investigated by our group.

Pathology	HIP (EEC: Salmon)	PCX (EEC: Purple)	TCX (EEC: Blue)
**Aβ IHC**	N/A	N/A	N/A
**$Aβ_{1\text{-}40}$ (pg/mg)**	N/A	N/A	N/A
**$Aβ_{1\text{-}42}$ (pg/mg)**	N/A	Green Yellow (t=-2.995, p=0.00376)	N/A
**$Aβ_{1\text{-}42}$ /$Aβ_{1\text{-}40}$**	Green (t=-2.813, p=0.00639)	N/A	N/A
**AT8 IHC**	Dark Grey (t=-3.021, p=0.0035)	N/A	N/A
**P-Tau (ng/mg)**	Orange (t=-3.681, p=0.000458)	Green (t=-3.209,p=0.00201)<br>Purple (t=-3.681,p=0.000453)	Brown (t=-3.041,0.00329)<br>Black (t=-4.601,p=1.76e-05)<br>Pink (t=3.110,p=0.00268)
**P-Tau/Tau_{total}**	Dark Grey (t=-2.703, p=0.00864)<br>Orange (t=-3.533, p=0.000739)	Green (t=-2.788,p=0.00683)<br>Purple (t=-3.331 ,p=0.00139)	Brown (t=-2.865,0.00558)<br>Black (t=-3.994,p=0.000166)

Supplementary Table 3: Associations between consensus modules and pathology indicators. t-statistics and associated p-values for the correlation between eigengenes and pathology values for the modules generated in the consensus WGNCA. The modules that demonstrated significant associations are listed with relevant statistical values. In the header for each region, the module that represents the “endfoot enriched cluster” (EEC) is listed. If no significant associations were found N/A is listed.

Region	Dementia Status	*AQP4*	*DTNA*	*DMD*	*DAG1*	*SNTA1*
**Hippocampus**	**All**	24.31132	8.89647	53.87267	11.78569	15.0304
**Hippocampus**	**Demented**	21.95324	8.005868	28.3778	13.77576	16.53115
**Hippocampus**	**Non-Demented**	19.97146	17.24965	71.00481	8.265678	10.5473
**Hippocampus**	**Consensus**	17.52429	11.19295	14.52311	6.287166	8.333624
**PCX**	**All**	6.369115	8.089588	0.164347	6.723977	6.202443
**PCX**	**Demented**	6.667535	4.20171	0.266834	5.742807	3.070595
**PCX**	**Non-Demented**	9.910242	5.15381	2.152283	2.141125	2.911557
**PCX**	**Consensus**	7.436407	6.646487	5.805066	8.3311	10.8394
**TCX**	**All**	10.61677	13.74225	7.926699	1.462676	6.831924
**TCX**	**Demented**	6.054626	5.093205	4.274095	0.28934	4.064454
**TCX**	**Non-Demented**	11.79898	15.38296	1.1218	5.384408	11.05665
**TCX**	**Consensus**	10.00701	8.353792	3.238163	2.980986	4.036345

Supplementary Table 4: Intramodular connectivity values for DAC-complex genes. Intramodular connectivity was derived for each of the DAC genes, within each of the WGCNA runs that was completed, including single network WGCNA for all subjects as well as demented and non-demented independently. Additionally, the intramodular connectivity values are included for the consensus modules that were generated.

Supplementary Datasets

(Attached separately as excel spreadsheet)

Supplementary Dataset 1: Complete list of WGCNA-based clustering results. The clustering assignments for all genes included in the WGCNA analysis for each brain region. The clusters generated for both the single-network WGCNA and the consensus WGCNA are included. Frontal white matter was excluded due to the lack of co-clustering of DAC genes. The "endfoot enriched" clusters are denoted in the region key.
